# Residual Symptoms and Quality of Life After Treated Lyme Neuroborreliosis: Case-Control Study (QoLYME)

**DOI:** 10.1093/ofid/ofaf042

**Published:** 2025-01-24

**Authors:** Julie Foret, Anne-Julie Paren, Souheil Zayet, Catherine Chirouze, Vincent Gendrin, Kevin Bouiller, Timothée Klopfenstein

**Affiliations:** Department of Infectious and Tropical Diseases, CHU Besançon, Besançon, France; Department of Infectious and Tropical Diseases, Nord Franche-Comté Hospital, Trevenans, France; Department of Infectious and Tropical Diseases, Nord Franche-Comté Hospital, Trevenans, France; Department of Infectious and Tropical Diseases, CHU Besançon, Besançon, France; Université Marie et Louis Pasteur, CHU Besançon, CNRS, Chrono-environnement (UMR 6249), F-25000 Besançon, France; Department of Infectious and Tropical Diseases, Nord Franche-Comté Hospital, Trevenans, France; Department of Infectious and Tropical Diseases, CHU Besançon, Besançon, France; Université Marie et Louis Pasteur, CHU Besançon, CNRS, Chrono-environnement (UMR 6249), F-25000 Besançon, France; Department of Infectious and Tropical Diseases, Nord Franche-Comté Hospital, Trevenans, France

**Keywords:** fatigue, Lyme borreliosis, Lyme neuroborreliosis, quality of life, residual symptoms

## Abstract

**Background:**

Earlier studies revealed that 10%–50% of patients reported remaining complaints after treatment for Lyme neuroborreliosis (LNB). The aim of our study was to assess symptoms and quality of life in patients with diagnosed and treated LNB and to compare them with findings in the general population.

**Methods:**

Adults with LNB receiving adequate antibiotics were included between 2015 and 2021 in 2 tertiary hospitals. Two controls without Lyme borreliosis history were included for each case patient, matched by age and geographic area. All participants were interviewed to answer a standardized questionnaire. Fatigue was assessed by the Fatigue Severity Scale (FSS) and quality of life by the 12-Item Short Form Survey, including physical component summary (PCS) and mental component summary (MCS) scores.

**Results:**

Fifty-three patients and 104 controls were included. The mean age (SD) was 62 (13) years in both groups; 66% were male in the LNB group and 44% in the control group (*P* = .01). Fatigue (68% vs 48%, respectively; *P* = .02), memory disorders (60% vs 38%; *P* < .01), and attention disorders (32% vs 17%; *P* = .05) were significantly more frequent in the LNB group than in controls. In multivariable analysis, no association was found between LNB and FSS scores (odds ratio, 1.6 [95% confidence interval, .9–3.0]; *P* = .15) or between LNB and MCS scores (0.8 [.4–1.5]; *P* = .45); however, patients with LNB had lower PCS scores (0.5 [.3–.9]; *P* = .03).

**Conclusions:**

Several symptoms were similar in patients with LNB and controls. Quality of life was slightly impaired in patients with LNB and PCS scores were lower, but there were no differences in MCS or FSS scores. Reassurance and specific rehabilitation measures could be provided to these patients.

## Graphical Abstract

**Graphical Abstract:**
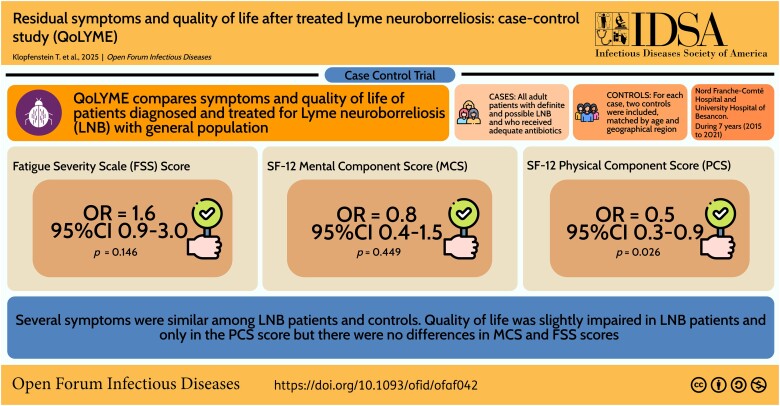
This graphical abstract is also available at Tidbit: https://tidbitapp.io/tidbits/residual-symptoms-and-quality-of-life-after-treated-lyme-neuroborreliosis-case-control-study-qolyme-5384b4bb-4411-41dd-8e90-4ffcc7f6ef50

Lyme borreliosis (LB) is a tick-borne disease caused by spirochetes of the *Borrelia burgdorferi* sensu lato complex and transmitted, mainly in Europe, by the tick *Ixodes ricinus* [1]. LB is the most common tick-borne disease in the Northern hemisphere. In 2022, according to the French sentinel network report, the incidence rate was estimated 51 cases per 100 000 inhabitants in France, with large regional disparities [2, pp 90–96].

Lyme neuroborreliosis (LNB) is the second most common manifestation in France [3] after erythema migrans (EM). LNB occurred in 10%–20% of LB cases, during the early phase of the disease (sometimes with concomitant EM) or after several weeks or even months following the tick bite [4]. Early LNB consists of meningoradiculitis, also known as Bannwarth syndrome at the spinal level, expressed by atypical radicular pain that is resistant to usual analgesics, leading to insomnia. Headache and sensory or motor disorders are rarely observed. Confirmed diagnosis of LNB includes compatible clinical presentation with lymphomonocytic cell pleocytosis and intrathecal Borrelia antibody production [4]. After accurate antibiotic treatment (with intravenous ceftriaxone or oral doxycycline for 14–21 days, depending on the duration of manifestations), most of the symptoms resolve [5–7].

However, 10%–50% of patients report remaining symptoms related to LNB, months after treatment [5, 8–10], such as radicular or muscular pain, arthralgia, fatigue, headache, cognitive impairment and paresthesia. Posttreatment Lyme disease syndrome is defined by the following inclusion criteria: (1) a documented episode of LB in an adult or child; (2) resolution or stabilization of the objective manifestation(s) of LB after treatment of the episode; (3) onset of definite subjective symptoms within 6 months after the diagnosis of Lyme disease and persistence for ≥6 months after antibiotic therapy; and (4) subjective symptoms resulting in a substantial reduction in occupational, educational, social, or personal activities [11]. Although posttreatment Lyme disease syndrome is currently well recognized by most experts, it still a matter of debate given the high rate of similar symptoms in the general population [12–16]. Its pathogenicity is poorly understood. The predominance of residual symptoms is subjective and influenced by the association of various demographic and clinical characteristics (age, sex [9], cognitive-behavioral factors [17], underlying neuropsychiatric conditions [18], and delays in antimicrobial treatment [19]), coinfection with other tick-borne pathogens, and microbiological, immunological [9], and genetic factors.

These residual symptoms increase the number of outpatient consultations [20] and impair quality of life [21, 22]. It is important to recognize the symptoms and identify the underlying mechanisms contributing to their development.

In a retrospective multicenter study in France, Naudion et al [10] found that 60% of patients with LNB reported residual symptoms during their follow-up (median duration of follow-up [interquartile range (IQR)], 70 days [30–175]) [10]. The most frequently reported manifestations included radicular pain (25%), paresthesia (13%), and fatigue (12%). However, we cannot be certain that these symptoms were related to LNB and differed from those reported in the general population. The aim of our study was to assess symptoms and quality of life in patients with diagnosed and treated LNB, compared with a population control group.

## MATERIAL AND METHODS

### Study Design

QOLYME is an observational prospective case-control study conducted in Franche-Comté region, France, a highly endemic region of LB (incidence, 152 [66–238]/100 000 inhabitants in 2022) [2, pp 90–96].

### Study Population

Case patients were outpatients or hospitalized patients with an LNB diagnosis in Nord Franche-Comté Hospital or the University Hospital of Besançon, between January 2015 and December 2021. A diagnosis of LNB was defined as definite or possible LNB; all patients had symptoms compatible with LNB and one or both cerebrospinal fluid (CSF) analysis criteria (CSF pleocytosis and/or antibody index indicating intrathecal anti-*Borrelia* antibody synthesis) [4].

The study included all adult patients (aged ≥18 years) who had definite or possible LNB and who received adequate antibiotics (according to French scientific societies’ guidelines on LB and other tick-borne diseases [4]). For each case patient, 2 controls were included, matched by age (±10 years) and geographic region (mainly in the family or in the neighborhood of the case patient). Control patients were recruited with the participation of case patients and were excluded if they had a history of LB (including EM) or a tick bite within the last 5 years.

### Questionnaire and Outcome Measures

All participants were interviewed by phone (from January to July 2022) and were asked to respond to a standardized questionnaire including medical history, exposure to ticks, and the presence of symptoms within the last month (pain, paresthesia, fatigue, headache, weight loss, joint pain, muscle pain, sleeping disorders, concentration/memory difficulties, irritability, dizziness, ocular symptoms, or nausea). Medical history, including psychiatric history (depression or anxiety disorders), was retained if a physician had mentioned the diagnosis to the patient. Neurological pain was defined as a pain with neurogenic features, such as burning and/or electrical discharges and/or associated paresthesias. Fatigue was assessed by the Fatigue Severity Scale (FSS), a scale that includes 9 items, each with a score ranging from 1 to 7 (7 being the worst) [23].

The self-reported health-related quality of life was calculated with the 12-Item Short Form Survey (SF-12), the short version of the 36-Item Short Form Survey (SF-36), which is indexed into 2 components: the physical component summary (PCS) and the mental component summary (MCS) scores [24]. Index scores range from 0 to 100, where a higher score indicates better physical or mental health–related quality of life. We also calculated the Charlson Comorbidity Index (CCI) [25].

### Ethical Approval and Patient Consent

This study was approved by the local ethics committee, Nord Ouest II, Amiens, France (national no. 2022-A01583-40). All participants provided written informed consent.

### Statistical Methods

We expressed discrete variables as numbers and percentages and continuous variables as means, SDs, and 95% confidence intervals (CIs). Medians and IQRs were used instead of mean and SDs for skewed distributions. Comparisons between patients with or without LNB were performed using bivariate logistic regression (Wald test for variables with 2 modalities and likelihood ratio test for multimodal variables) to take into account the benefit of case-control methodology.

The multivariable analysis model chosen was an ordinal logistic regression model. The variables included variables to be forced (variables retained as potential confounders on literature data): LNB (main explanatory variable), age, sex, CCI, cancer, obesity, and fibromyalgia or chronic fatigue syndrome. For the SF-12 score, the rheumatological history and psychiatric disorders were also selected as variables for the physical and psychological components, respectively. For the other comorbid conditions, we also selected variables with *P* values <.20, based on the results of the bivariate analysis.

We used a significance level of *P* < .05 and performed all analyses using R software, version 4.2.1 (R Project for Statistical Computing; https://www.r-project.org). Concerning the goodness-of-fit test for ordinal response models, Hosmer-Lemeshow (for the ordinal model), Lipsitz, and Pulkstenis-Robinson (χ^2^ and deviance) tests were performed.

## RESULTS

### Patient Characteristics and Univariate Analysis

**Figure 1.**
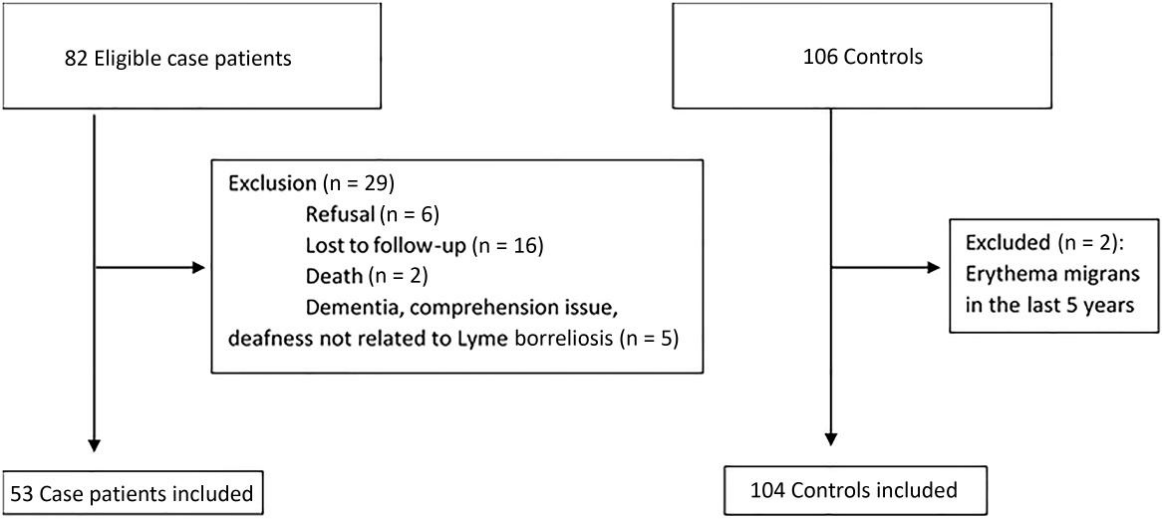
Flow chart of patient enrollment in the QOLYME study, Franche-Comté Region, 2015–2021.

Between January 2015 and December 2021, 82 patients with a diagnosis of definite or possible LNB were eligible for our study. Among these eligible patients, 29 were excluded, due to refusal to participate (n = 6), no answer/loss to follow-up (n = 16), death (n = 2), or other reasons, such as dementia, comprehension issues, or deafness (n = 5). Finally, we included 53 case patients and 106 matched controls (Figure 1). Two controls were excluded a posteriori because of a history of EM in the last 5 years. All case patients received antibiotics in accordance with European recommendations, with ≥14–21 days of doxycycline (200 mg/d orally) or intravenous ceftriaxone (2 g/d) [4]. The median time (IQR) between lumbar puncture and questionnaire completion was 55 (21.9) months.

The mean age was 62 years in both groups; 66% were male in the LNB group and 44% in control group (*P* = .01). Sixty-two percent of patients overall (99 of 157) had ≥1 comorbid condition. Neurological comorbid conditions was present in 19% of LNB case patients and in only 8% of controls (*P* = .051) (Table 1). Only 4% of case patients and 2% of matched controls had a history of fibromyalgia or chronic fatigue syndrome (*P* = .6). All patients with LNB reported a history of ≥1 tick bite during their life, and only 30% reported an EM.

**Table 1.** Characteristics of Patients With Lyme Neuroborreliosis and Controls

Characteristic	Patients, No. (%)^a^			*P* Value^b^
	Overall (n = 157)	LNB (n = 53)	Controls (n = 104)	
Male sex	81 (52)	35 (66)	46 (44)	.01
Age, years, mean (SD), y	62.4 (13.6)	62.8 (13.2)	62.1 (13.8)	.15
BMI, mean (SD)^c^	25.6 (4.9)	25.6 (4.3)	25.5 (5.2)	>.9
Tobacco consumption	23 (15)	9 (17)	14 (13)	.6
Arterial hypertension	51 (32)	15 (28)	36 (35)	.4
Cardiovascular^d^	30 (19)	6 (11)	24 (23)	.085
Neurological history^e^	18 (11)	10 (19)	8 (8)	.051
Pulmonary history^f^	22 (14)	9 (17)	13 (12)	.5
Diabetes mellitus	19 (12)	7 (13)	12 (12)	.7
Rheumatological history^g^	37 (24)	10 (19)	27 (26)	.3
Solid and/or hematological cancer	17 (11)	7 (13)	10 (10)	.5
Psychiatric history^h^	23 (15)	8 (15)	15 (14)	>.9
CFS-fibromyalgia	4 (3)	2 (4)	2 (2)	.6
High tick exposure^i^	77 (49)	34 (64)	43 (41)	.03
History of tick bites	90 (57)	53 (100)	37 (36)	<.001
No. of tick bites				
1–5	68 (76)	37 (70)	31 (84)	.1
5–10	10 (11)	6 (11)	4 (11)	
>10	12 (13)	10 (19)	2 (5)	
Time since last tick bite				
<1 y	11 (12)	11 (21)	0 (0)	<.001
1–5 y	26 (29)	26 (49)	0 (0)	
>5 y	53 (59)	16 (30)	37 (100)	
History of EM	16 (18)	16 (30)	0 (0)	…
Time from lumbar puncture to questionnaire, median (IQR), mo	…	55 (21.9)	…	…

Abbreviations: BMI, body mass index; CFS, chronic fatigue syndrome; EM, erythema migrans; IQR, interquartile range; LNB, Lyme neuroborreliosis.

^a^Data represent no. (%) of patients unless otherwise specified.

^b^ *P* values calculated by means of a bivariate logistic regression model (Wald test for variables with 2 modalities and likelihood ratio test for multimodal variables).

^c^BMI calculated as weight in kilograms divided by height in meters squared.

^d^Cardiovascular history include atrial fibrillation, heart failure, coronary artery disease, obliterative arterial disease of the lower limbs, carotid stenosis, and valvular heart disease.

^e^Neurological history include stroke, transient ischemic attack, Parkinson disease, multiple sclerosis, Alzheimer disease, meningioma, facial paralysis, diabetic sensory neuropathy, cruralgia with narrow lumbar canal, pudendal neuralgia, restless legs syndrome, and migraine.

^f^Pulmonary history includes chronic obstructive pulmonary bronchitis, asthma, and sleep apnea syndrome.

^g^Rheumatological history includes rheumatoid arthritis, rheumatoid pseudopolyarthritis, hip or knee prosthesis, fracture (vertebra, lower or upper limb), herniated disk, osteoarthritis, scoliosis, spondylolisthesis, carpal tunnel, and shoulder/knee tendon or ligament lesion.

^h^Psychiatric history includes depression and irritability.

^i^Walks in the woods/undergrowth and regular gardening (at least twice a month from May to October).

**Figure 2.**
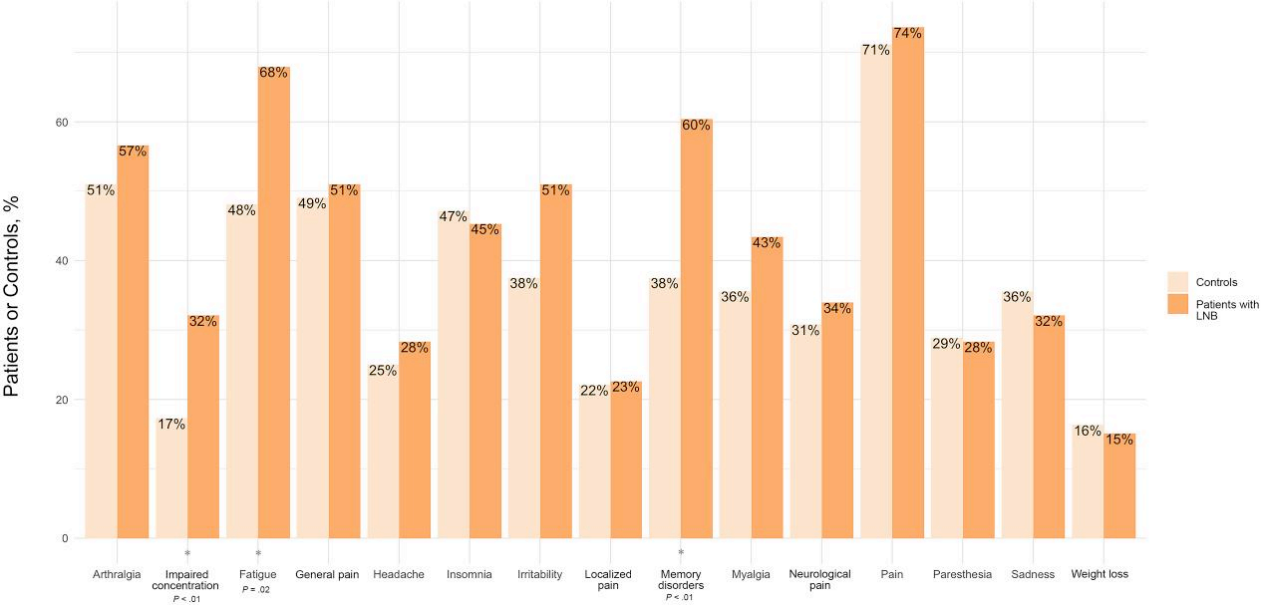
Distribution of symptoms in patients with Lyme neuroborreliosis (LNB) and controls.

The most common symptom in both groups was pain (74% in the LNB vs 71% in the control group; *P* = .8), including either localized or general pain (23% vs 22% [*P* = .9] and 51% vs 49% [*P* = .9], respectively), neurological pain (34% vs 31%; *P* = .7), myalgia (43% vs 36%; *P* = .4), arthralgia (57% vs 51%; *P* = .5), and headache (28% vs 25%; *P* = .6) (Figure 2). The symptoms that differed significantly between the 2 groups were fatigue (68% in LNB case patients vs 48% in controls; *P* = .02), memory disorders (60% vs 38%; *P* < .01), and attention difficulties (32% vs 17%; *P* = .05).

**Figure 3.**
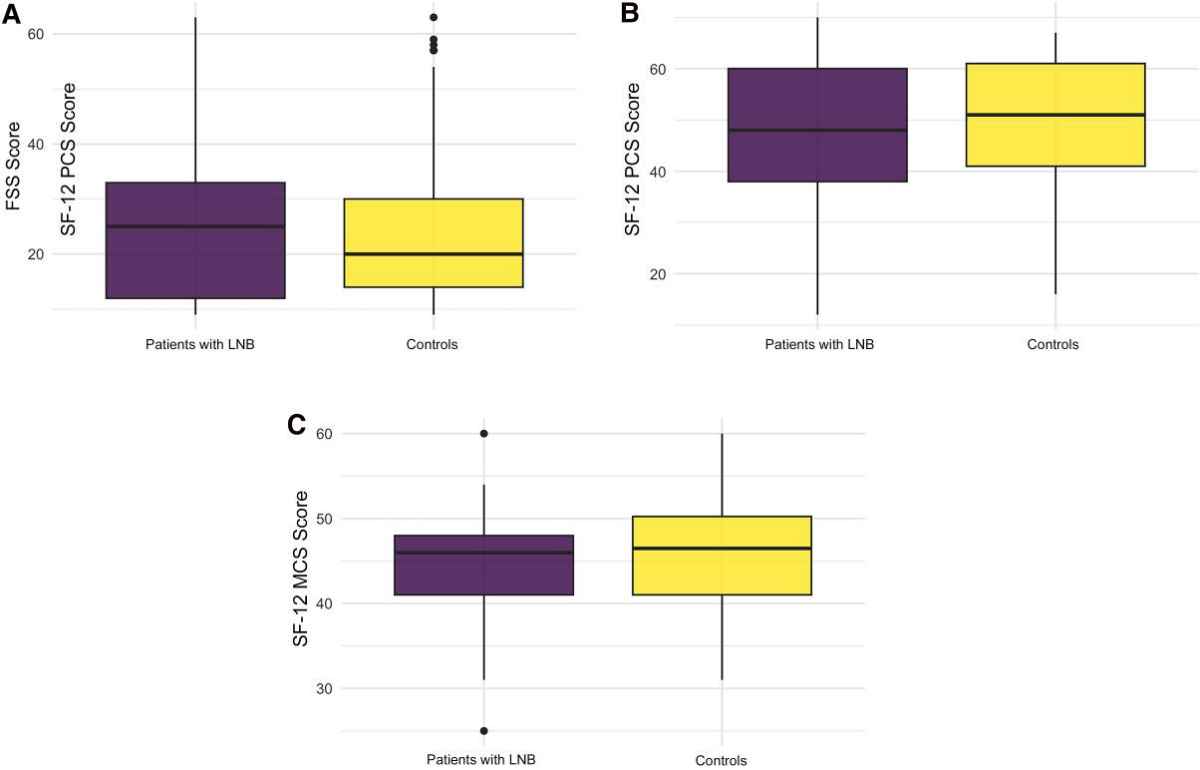
Comparison of Fatigue Severity Scale (FSS) score and the 12-Item Short Form Survey (SF-12) score between patients with Lyme neuroborreliosis (LNB) and controls (*P* = .2). Quartile diagrams show the distribution of FSS score (*A*), SF-12 physical component summary score (PCS) (*B*), and SF-12 mental component summary (MCS) score (*C*).

The mean FSS score was slightly higher in LNB case patients than in controls (27.5 [SD, 16.9] vs 23.9 [13.3]), not a significant difference (*P* = .2). Similarly, the 2 components (physical and mental) of the SF-12 score were slightly but not significantly lower in case patients (mean [SD], 47 [15] vs 50 [13] for physical and 44.6 [6.4] vs 46.1 [6.4] for mental; both *P* = .2 ) (Figure 3).

### Multivariable Analysis

**Figure 4.**
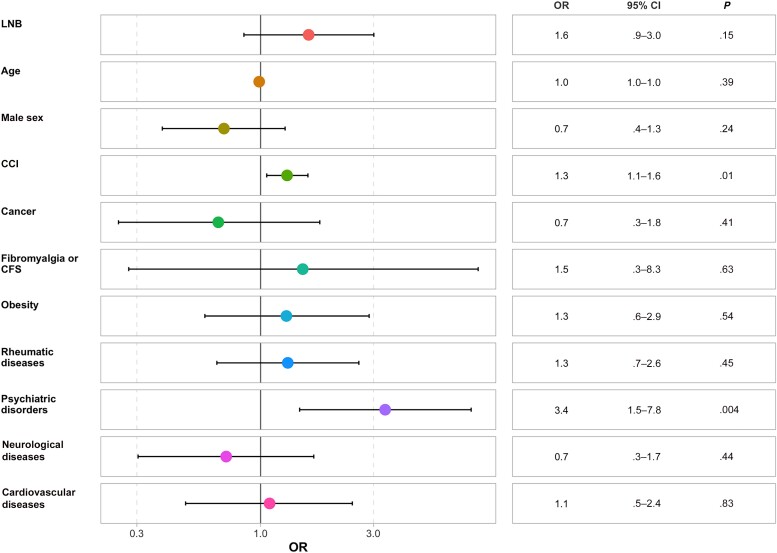
Forest plot of the Fatigue Severity Scale. Abbreviations: CCI, Charlson Comorbidity Index; CFS, chronic fatigue syndrome; CI, confidence interval; LNB, Lyme neuroborreliosis; OR, odds ratio.

The history of LNB did not affect the FSS score independently of sex, age, and comorbid conditions (odds ratio [OR], 1.6 [95% CI, .9–3.0]; *P* = .15) (Figure 4). We reported only 2 independent predictors of a higher FSS score: psychiatric history (OR, 3.4 [95% CI, 1.5–7.8]; *P* = .004) and CCI (1.3 [1.1–1.6]; *P* = .01).

**Figure 5.**
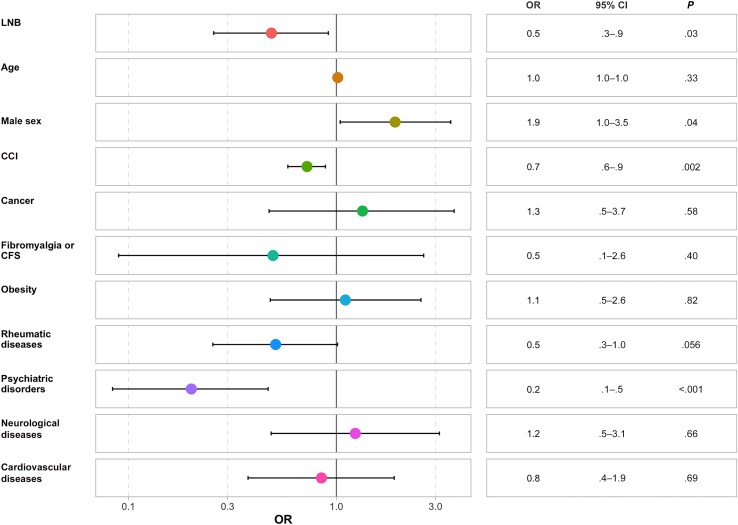
Forest plot of the physical component summary score of the 12-Item Short Form Survey (SF-12). Abbreviations: CCI, Charlson Comorbidity Index; CFS, chronic fatigue syndrome; CI, confidence interval; LNB, Lyme neuroborreliosis; OR, odds ratio.

**Figure 6.**
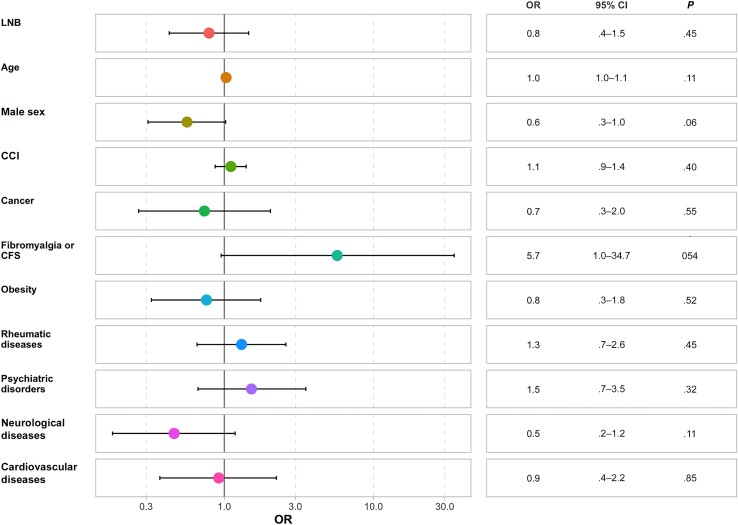
Forest plot of the mental component summary score of the 12-Item Short Form Survey (SF-12). Abbreviations: CCI, Charlson Comorbidity Index; CFS, chronic fatigue syndrome; CI, confidence interval; LNB, Lyme neuroborreliosis; OR, odds ratio.

Patients with LNB had a lower PCS score independently of sex, age, and comorbid conditions (OR, 0.5, [95% CI, .3–.9]; *P* = .03) (Figure 5). Psychiatric history (OR, 0.2 [95% CI, .1–.5]; *P* < .001) and CCI (0.7 [.6–.9]; *P* = .002) were independent predictors of the PCS score (Figure 6).

No association was found between LNB and MCS scores (OR, 0.8 [95% CI, .4–1.5]; *P* = .45) and no significant difference was found between the different variables and MCS scores. Hosmer-Lemeshow, Lipsitz, and Pulkstenis-Robinson tests indicated a good correlation between predicted and observed values (with all *P* values >.2 and most near 1) ,which demonstrate good predictability in the different models (Supplementary Table 1 and Supplementary Data).

## DISCUSSION

Most patients presented with symptoms regardless a history of LNB. Only fatigue, memory disorders, and attention difficulties were significantly more frequent in patients with LNB (univariate analysis). However, quality of life was impaired slightly in the physical component of SF-12, while the MCS and FSS scores were not associated with a history of LNB.

### Symptoms

Pain was the most frequent symptom, occurring in about three-quarters of patients and without a difference between patients with LNB and controls. The presence of symptoms was self-reported, without the use of a validated scoring system.

Residual symptoms after LNB are more frequent when the diagnosis is uncertain and are subject to debate, with conflicting findings [10, 15]. In the study of Naudion et al [10], among 138 patients included (72 with definite and 59 with possible LNB), 59% had residual symptoms, mostly radicular pain (25%). However, these findings were probably overestimated, because only 10% of patients had 1-year follow-up. Moreover, the median duration of follow-up (IQR) was 70 (30–175) days, shorter than in our study (55 [21.9] months) and different among patients with LNB. Compared with our study, patients were younger, and a majority were men.

In a prospective cohort study, Ursinus et al [8] compared persistent symptoms in patients with disseminated LB (n = 58) with those in controls from the general population (n = 1942), matching by age, sex, geographic region, and month of enrollment. Fatigue and pain were the most common symptoms in both groups and were more frequent in the disseminated LB group than in controls (29.2% vs 16.3%, respectively, for fatigue [*P* ≤ .05] and 20.3% vs 5.3% for pain [*P* ≤ .001]), which is consistent with our results concerning fatigue.

In our study, memory disorders and attention difficulties were more frequently observed in the LNB group in univariate analysis. Data from a Norwegian study were similar: 46% of patients with LNB (n = 50) described memory problems versus 10% of controls (n = 50) without a history of LNB and matched for age, education level, and sex (*P* < .001). In addition, 34% of patients with LNB reported concentration impairment versus 8% of controls (*P* = .003) [21]. Cognitive impairment in LNB has been extensively studied. Several findings have demonstrated the presence of cognitive disorders using validated tests, especially deficits affecting memory process [26–29]. Benke et al, in a cohort of 20 patients, reported long-term neurocognitive deficits, including reduced verbal learning, memory, and attention/executive function, several years after LNB (average interval, 51.6 months) [30].

Contrary to these results, some studies found no reduced cognitive function in patients with LNB. According to Andreassen et al [31, 32], the prognosis regarding cognitive functions was favorable, using validated neuropsychological tests (WMS-III, WAIS-IV, and D-KEFS). No difference was found in cognitive functions between the LNB group (n = 72) and controls (n = 68) at baseline or 6 and 12 months after treatment, and only 14 patients with LNB were lost to follow-up at 12 months. Similarly, Dersch et al [15], in a case-control study, found no difference between patients with definite LNB and controls 4 years after treatment, using the Mini-Mental Status Examination (MMSE) and Verbal Learning and Memory Test (VLMT). However, the MMSE is a screening test used to detect cognitive disorders in elderly patients and may be less precise than other neuropsychological tests.

In our study, we hypothesized that cognitive problems could have been overestimated in patients with LNB for several reasons: the high frequency of neurological comorbid conditions, the mean age of these patients, and, particularly, the self-reporting method. All patients with LNB had a tick history, which is not consistent with the literature, since tick bites are generally found in 30%–40% of cases [33]. This is due to the formulation of the question regarding tick bites, which asked only about exposure (≥1 bite in the past) and not about a tick bite before LNB.

### Fatigue and FSS Score

The FSS scores in patients with LNB were moderately higher than in controls, but not significantly so. While fatigue was more frequent in the LNB group, there was no significant association between LNB and the FSS score, independently of sex, age, and comorbid conditions. Our findings are in contrast with those from the case-control study of Eikeland et al [21]. They reported more fatigue in the LNB group than in control patients (mean FSS score [SD], 3.5 [2.4] vs 2.1 [1.4], respectively; *P* < .001). Moreover, 50% of LNB case patients reported fatigue as a daily problem, compared with 16% of controls (*P* = .001). The study by Eikeland et al was the first European study to focus on quality of life using SF-36, with lower PCS and MCS scores in patients with LNB than in controls (mean PCS score [SD], 44 [9] vs 51 [6], respectively [*P* < .001]; mean MCS score, 49 [11] vs 54 [6] [*P* = .01]). However, these findings should be interpreted with caution. The case definition was not strict, as one-third of patients were classified as having possible LNB (with neurological symptoms suggestive of LNB and ≥1 of the following criteria: pleocytosis, IAI, *Borrelia* antibodies in serum, and verified EM during the past 4 months). Similarly, a more recent Norwegian study reported higher levels of fatigue in the LNB group than in controls 6 months after treatment (mean FSS score [SD], 3.8 [1.7] vs 2.9 [1.3], respectively; *P* = .001) [32].

To assess the prevalence of fatigue, the FSS is the most commonly used score. Depending on the study, it can be expressed in different ways, either as the sum of all items (ranging from 9 to 63) [22] or as the mean of the 9 items (ranging from 1 to 7) [14, 21]; therefore, comparisons between studies can be more challenging. Furthermore, other standardized scores may be used, such as the Modified Fatigue Impact Scale [34], initially developed to assess fatigue in patients with multiple sclerosis [35].

Fatigue is not a specific symptom of LNB and can result from other infections, such as *Brucella* spp, Epstein-Barr virus [36], or *Coxiella burnetii* infections [37]. While in our study a history of LNB did not affect the FSS score, other findings suggest that the severity of acute illness was predictive of residual fatigue [36]. In additions, other persistent symptoms have been reported in other diseases, such as coronavirus disease 2019 (COVID-19) [38], chikungunya [39], or dengue [40], suggesting the presence of a general postinfectious syndrome or other underlying mechanisms. However, the inclusion period of our study allows us to eliminate alternative diagnoses, such as long COVID or postacute sequelae of severe acute respiratory syndrome coronavirus 2 infection [41].

### Quality of Life Score

Patients with LNB had a moderately lower health-related quality of life, but only on the PCS score. The physical impairment in quality of life in patients with LNB should be put in perspective. In fact, history of LNB reduced the PCS by 1 point, independently of sex, age, and comorbid conditions, with an OR of 0.5 (95% CI, .3–.9), from a possible total score of 70. Moreover, the MCS score did not differ between case patients and controls.

In the case-control study of Dersch et al [15], lower PCS and MCS scores were reported but not significantly lower. However, in a subgroup analysis comparing patients with LNB with (n = 17) or without (n = 13) residual symptoms, they found significantly lower PCS in patients with residual symptoms (mean [SD], 46.33 [8.38] vs 57.38 [3.04] in those without residual symptoms; *P* < .001), but no difference in MCS score. These data strengthen the idea that the presence of residual symptoms may negatively affect patients’ quality of life.

In our study, the presence of psychiatric history and other comorbid conditions were factors that could influence patients’ physical quality of life. Other authors have investigated potential determinants of residual symptoms in patients with LNB. An American study highlighted that having comorbid conditions was associated with lower PCS score in patients with LNB at baseline (mean [SD], 42.7 [10.4] vs 50.1 [9.6] in those without comorbid conditions; *P* = .009) [42], which is in line with our results.

The majority of studies compared patients with LNB and a control group, mostly healthy people, but it can remain challenging to evaluate results because control populations may differ. Indeed, our control group presented more fatigue than controls included in the study of Rebman et al [22] (mean FSS score [SD], 23.9 [13.3] vs 19.8 [8.6], respectively). These discordant findings could be explained by 2 factors. First, in that American study, controls were younger than in our study (mean age, 54.7 vs 62.1 years); second, LNB case patients and controls had fewer comorbid conditions than our overall population, as cancer, fibromyalgia, or chronic fatigue syndrome were exclusion criteria. In addition, MCS and PCS scores were lower in our control group (mean [SD], 46.1 [6.4] for MCS and 50 [13] for PCS) than in the literature (54.2 [5.4] and 55.1 [6.2], respectively, for Rebman et al [22] and 51.77 [8.79] and 54.72 [5.48] for Dersch et al [15]).

Finally, when considering overall patient outcomes, including our findings, studies are mostly reassuring, with favorable long-term prognosis after antibiotic treatment [14, 42–44]. The discordant results we have previously described could be explained, among other factors, by the use of different study designs, various case definitions, and different durations of follow-up.

### Strengths and Limitations

The strength of our study lies in its case-control design (with 2 controls per case patient). Case-control studies can assess correlation with several symptoms, which is useful for LNB. To limit the risk of confusion bias, we performed a multivariate analysis. To limit bias that could affect the study results, we used a telephone conversation rather than a self-report questionnaire. The telephone interview was short (an average of 20 minutes), and standardized questionnaires (FSS and SF-12) were also used.

One limitation of our study is the small sample size of patients with LNB, which could have made it difficult to detect some differences between the 2 groups of patients due to a lack of statistical power; however, the 1:2 case-control design could limit this impact. Furthermore, a serological test for LB was not required in control subjects to definitively rule out the possibility of this disease; however, no history of LB (including EM) and the absence of a tick bite in the last 5 years reduced the risk of undiagnosed LB. CSF findings were included in our study criteria, unlike the CDC criteria, which do not require CSF findings for LNB diagnosis; this could also explain the differences found between populations in the literature. Another limitation of our study is the presence of a heterogeneous patient group, including definite and possible LNB cases, as well as variations in the duration of follow-up between lumbar puncture and completion of the questionnaire.

### Conclusions

Considering these findings, it is crucial to reassure patients that LNB has a low impact on quality of life compared with a control population. Being able to say that the persistence of symptoms is not necessarily greater than that in a control population is a way to prevent anxiety generated by an LNB diagnosis. Specific rehabilitation measures could be suggested in patients with fatigue and pain. Furthermore, it is essential for practitioners to remain objective and always seek differential diagnoses for nonspecific symptoms reported by patients following LNB.

## Supplementary Data

Supplementary materials are available at *Open Forum Infectious Diseases* online. Consisting of data provided by the authors to benefit the reader, the posted materials are not copyedited and are the sole responsibility of the authors, so questions or comments should be addressed to the corresponding author.

## Supplementary Material

ofaf042_Supplementary_Data
