# Supplementary material for: Residual Symptoms and Quality of Life After Treated Lyme Neuroborreliosis: Case-Control Study (QoLYME)
Source: Open Forum Infect Dis. 2025 Jan 24;12(2):ofaf042. doi: 10.1093/ofid/ofaf042 (PMC11842133; doi:10.1093/ofid/ofaf042)
Supplement: ofaf042_Supplementary_Data [file ofaf042_supplementary_data.docx]

**Supplementary data**

|  |  | Test value | Df | p-value |
| --- | --- | --- | --- | --- |
| FSS_T  model | Hosmer and Lemeshow test | 356.08 | 386 | 0.8603 |
|  | Lipsitz test | 10.778 | 9 | 0.2913 |
|  | Pulkstenis-Robinson chi-squared test | 2258.3 | 2528 | 1 |
|  | Pulkstenis-Robinson deviance test | 648.26 | 2528 | 1 |
| SF12_P model | Hosmer and Lemeshow test | 388.95 | 431 | 0.9277 |
|  | Lipsitz test | 0.87217 | 9 | 0.9997 |
|  | Pulkstenis-Robinson chi-squared test | 2588.2 | 2823 | 0.9993 |
|  | Pulkstenis-Robinson deviance test | 669.53 | 2823 | 1 |
| SF12_M model | Hosmer and Lemeshow test | 254.56 | 242 | 0.2771 |
|  | Lipsitz test | 6.9485 | 9 | 0.6425 |
|  | Pulkstenis-Robinson chi-squared test | 1398.4 | 1584 | 0.9997 |
|  | Pulkstenis-Robinson deviance test | 526.47 | 1584 | 1 |

**Table S1:** Goodness of fit tests for the ordinal logistic models.
